# The Detection of Early Epigenetic Inheritance of Mitochondrial Stress in *C. Elegans* with a Microfluidic Phenotyping Platform

**DOI:** 10.1038/s41598-019-55979-x

**Published:** 2019-12-17

**Authors:** H. B. Atakan, K. S. Hof, M. Cornaglia, J. Auwerx, M. A. M. Gijs

**Affiliations:** 1 Laboratory of Microsystems, Ecole Polytechnique Fédérale de Lausanne, CH-1015 Lausanne, Switzerland; 2 Laboratory of Integrative Systems Physiology, Ecole Polytechnique Fédérale de Lausanne, CH-1015 Lausanne, Switzerland

**Keywords:** Lab-on-a-chip, Biomedical engineering

## Abstract

Fluctuations and deterioration in environmental conditions potentially have a phenotypic impact that extends over generations. Transgenerational epigenetics is the defined term for such intergenerational transient inheritance without an alteration in the DNA sequence. The model organism *Caenorhabditis elegans* is exceptionally valuable to address transgenerational epigenetics due to its short lifespan, well-mapped genome and hermaphrodite behavior. While the majority of the transgenerational epigenetics on the nematodes focuses on generations-wide heritage, short-term and in-depth analysis of this phenomenon in a well-controlled manner has been lacking. Here, we present a novel microfluidic platform to observe mother-to-progeny heritable transmission in *C. elegans* at high imaging resolution, under significant automation, and enabling parallelized studies. After approximately 24 hours of culture of L4 larvae under various concentrations and application periods of doxycycline, we investigated if mitochondrial stress was transferred from the mother nematodes to the early progenies. Automated and custom phenotyping algorithms revealed that a minimum doxycycline concentration of 30 µg/mL and a drug exposure time of 15 hours applied to the mothers could induce mitochondrial stress in first embryo progenies indeed, while this inheritance was not clearly observed later in L1 progenies. We believe that our new device could find further usage in transgenerational epigenetic studies modeled on *C. elegans*.

## Introduction

Certain experiences and stress during the life of an organism can have significant effects on the successive generations^1^. Especially maternal stress can precede phenotypic alterations over the next generations^2^. Heritable phenotypic changes without alterations of the genotype are known as epigenetics^3^. For example, in humans, prenatal exposure to famine causes significant epigenetic changes resulting in adult disease risk^4^. This human epidemiological study showcases the need for understanding the effect of maternal/prenatal stressors on epigenetic modifications. These modifications are widely acknowledged as a crucial factor in the control of gene expression, influencing the phenotypes of multicellular organisms^5^. Various model organisms are used for understanding of complex biological pathways and their epigenetic regulations^6^, including plants, mice and *C. elegans*^7^. Although these model organisms show insight into the transmission of epigenetic information through the germline, the occurrence of this phenomenon in humans is inconclusive^8^. One of the main model organisms, *C. elegans*, offers the opportunity to study the mechanisms of transgenerational epigenetics due to its fully sequenced genome, well-studied genetics, near exclusive hermaphrodite population and short lifespan^9,10^. Therefore, a great deal of the research on the epigenetic inheritance has been performed on *C. elegans* as a model organism^11,12^. Up to three generations of nematodes originating from famished mothers generated increased lifespan^13^ and maternal food availability decreased progeny reproduction in food-sufficient environments^14^. Additionally, maternal age generated variations in the size at hatching, speed of development, growth rate and fecundity in the next generations^15^. Exposure of high glucose concentrations to *C. elegans* in the parental generation led to opposing effects on fertility, while providing protection against cellular stress in the descendent progenies^16^. Temperature-induced changes showed that for at least 14 generations, long lasting epigenetic memory of environmental change can be stored^17^. Deficiency in the chromatin modifiers in the parental generation extended the lifespan of the descendants up until the third generation^18^. Most of these works focused on long-term and generation-wide epigenetic inheritance.

Short-term analysis of the effects of stressed mothers on development during the embryonic and first larval stages is a field of epigenetic inheritance research that is perfectly compatible with microfluidics. The main challenge in the short-term epigenetic studies in *C. elegans* is to establish a sustainable approach to obtain sufficient data of both the parental generation and the progenies at high-resolution. Such procedures require assisting tools, like offered by microfluidics and Lab-on-a-chip (LoC) technologies, to accomplish both tasks at the same time instead of standard agar plate-based methods^19^. While early *C. elegans* epigenetics has not been explicitly studied in microfluidic chips, several platforms have been proposed which could have been suitable for studies of parental generation’s transition to the progenies. Combination of polydimethylsiloxane (PDMS)-based devices with agarose gels showed that single nematode resolution culture and first progeny tracking was possible, although these methods were laborious and a supply of a well-adjusted concentration and time profile in applying food or a drug could be problematic^20–22^. A microfluidic device with a built-in dissection board was designed to extract embryos from adult nematodes^23^. The relatively low-throughput and laborious protocols of such devices, however, may disfavor their usage. Worm development studies in worm culture chambers in PDMS devices at single or multiple animal resolution have also been proposed. While these devices could be adapted for progeny studies too, they employ complicated micro-fabrication protocols^24,25^ or they were designed to start the worm culture from the onset of L1 larval stage^26–28^, resulting in absence of data in the embryonic experimentation window. Alternative worm culture devices for controlled progeny evacuation^29^ or counting^30^ have also been demonstrated. However, considerable amount of modifications were required to enable high-resolution imaging and experimental parallelization. A PDMS design with embryo incubators has been proposed to observe embryogenesis of the first embryo progenies^31^. This design was further modified to obtain first embryo progenies by mechanically compressing adult nematodes^32^ or studying the development of embryos released from the parental generation in worm culture chambers^33^. Embryo incubators have been demonstrated to be ideal construction features for embryogenesis studies, but they lacked a mechanism to trap embryos permanently as embryos floated freely in the fluidic serpentines due to the natural shape of the incubators. A long-term adult worm trapping and embryo collection chamber has also been reported^34^. However, this device had a rather complicated micro-fabrication procedure and the identity of embryos inside the chambers was not conserved.

In this work, we present a microfluidic device for the observation of mother-to-progeny epigenetic inheritance in *C. elegans*. This new device provides impeccable trapping and identity tracking of a single *C. elegans* embryo at high-resolution and in a parallel fashion, in combination with statistical analysis. We provided significant automation in experimentation and post-experiment data analysis, empowering recording of high-content data from the mothers, embryos and L1 progenies. As mitochondria play a central role in epigenetic alterations^35^, epigenetic inheritance of mitochondrial stress in the early life stages of the progenies was selected as the platform’s validation study. A hallmark mitochondrial stress response is the mitochondrial unfolded protein response (UPR^mt^), a protective transcriptional response stabilizing the mitochondrial function triggered by a mitonuclear imbalance^36^. Using the UPR^mt^ reporter strain *hsp-6::gfp* allows to visualize mitochondrial stress, as this strain expresses green fluorescent protein (GFP) when mitochondrial function is destabilized. To mimic the mitonuclear imbalance and the following UPR^mt^ response, we have used doxycycline – an antibiotic drug that impairs mitochondrial proteostasis – at different concentrations and application conditions in order to investigate if epigenetic inheritance of the mitochondrial stress occurs. Our results revealed that a dose-dependent maternal nematode treatment resulted in mitochondrial stress in the embryos of the next generation indeed and that this inheritance could possibly be maintained in L1 progenies.

## Results

### Microfluidic chip design

**Figure 1:**
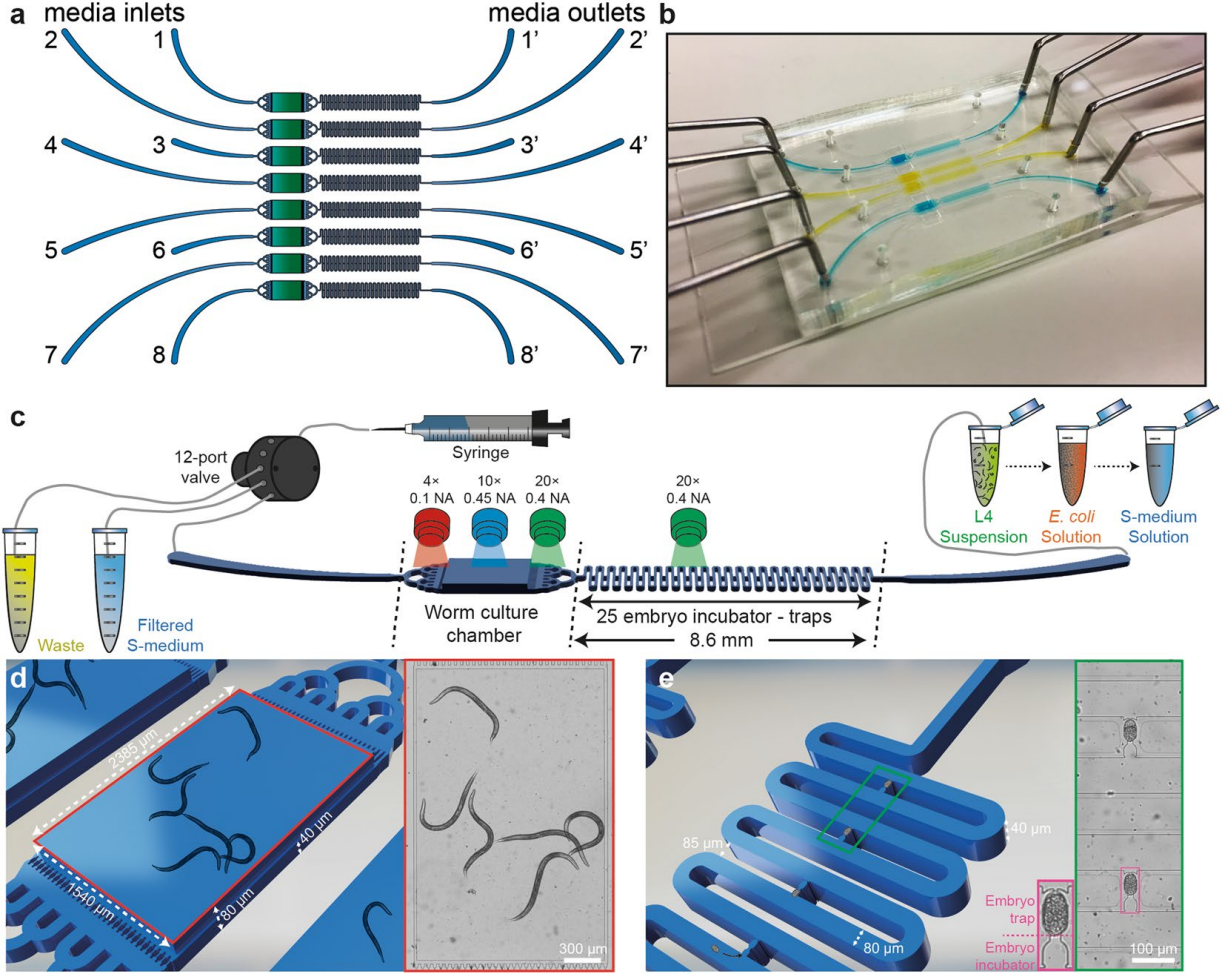
Details of the mother-to-progeny development platform for the automated phenotyping of the larvae and embryos of *C. elegans*. (**a**) Schematic drawing of the microfluidic device, which consists of eight lanes, each of which having a culture chamber for the worm development from the L4 larval stage to the adult stage and a serpentine with 25 embryo incubators and traps for the first progeny imaging. (**b**) Real-time image of the microfluidic chip, which is filled with liquid dye solutions. (**c**) Representation of the platform operation. A media inlet can be linked to a syringe pump, a waste and a S-medium reservoir through a 12-port valve. A media outlet can be coupled to a L4 suspension, an *E. coli* solution and a S-medium solution, respectively. While 4x, 10x and 20x objectives are used for the culture chamber imaging, a 20x objective only is used for embryogenesis imaging. (**d**) Three-dimensional (left) and real-time (right) images of a worm culture chamber with the key feature sizes marked. (**e**) Three-dimensional (left) and real-time (right) images of a serpentine with embryo traps and incubators labeled and feature sizes marked.

Our main design requirements were (i) to minimize the need of fluidic control operations, and (ii) to track the transition of the parental generation to its progenies. We established a microfluidic design that allows the loading and culture of the mother nematodes, and isolation and observation of the progenies. Our design consists of eight parallel microfluidic lanes, each lane having a single media inlet and a media outlet (Fig. 1a). We integrated a worm culture chamber in each microfluidic lane to culture nematodes from the L4 larval stage to the adult stage. A fluidic serpentine, which was designed to accommodate embryos after being laid from the mother nematodes, was also incorporated next to a worm culture chamber. The worm culture chamber has a size of 1540 µm × 2385 µm × 80 µm, which was experimentally tuned to accommodate up to 30 nematodes (Supplementary Fig. S1). The media inlet side of the growth chambers consists of 32 constriction filters with a width of 10 µm to prevent L1 progenies from escaping. The serpentine side of the growth chambers consists of 32 constriction filters with a width of 24 µm, which is tight enough to retain L4 larvae in the chamber and release embryos only upon a fluidic pressure increase from the worm culture chambers to the fluidic serpentines. A released embryo is located in one of the 25 embryo incubators and subsequently, forced to a tight confinement in the neighboring embryo trap. This microfluidic design is implemented in a PDMS part that has a size of 53 mm × 35 mm. A 75 mm × 38 mm glass microscope slide is used as the sealing part to cover the entire PDMS surface (Fig. 1b).

### Platform working principle

We used a 12-port syringe valve during the experiments. We reserved two ports of the 12-port valve as a backup (in case of a clogging in any of the other ports), and connected one port to a waste reservoir and another to an S-medium reservoir (Fig. 1c). Any waste generated during the worm and bacteria loading, was disposed in the waste reservoir. S-medium was supplied from its reservoir to transfer embryos out of the worm culture chambers and place them inside embryo traps. S-medium injection was also utilized to break any *E. coli* aggregate apart during the bacterial feeding of the nematodes. The remaining eight ports were linked to each media inlet of the microfluidic chip. This approach provided the maximum throughput that could be delivered with a 12-port valve. We relied on passive hydrodynamics – excluding any integration of on-chip components, such as pressure valves – during the course of the experimentation. The media outlets were interchangeably coupled to a L4 suspension, *E. coli* solution and bacteria-free S-medium solution reservoirs. While the L4 suspension contained L4 larvae suspended in S-medium solution, *E. coli* and S-medium solution reservoirs contained doxycycline at 0, 15, 30 or 60 µg/mL concentrations. Three different objectives – 4× (0.1 NA), 10× (0.45 NA) and 20× (0.4 NA) – were mounted on the setup and used, depending on the need of the experiment for high-resolution. A 4× objective was selected while acquiring time-lapse images of the mother nematode development in the worm culture chambers, as it completely covered the observation of a worm culture chamber (Fig. 1d). A 20× objective was chosen to record the physiological properties and fluorescent intensity expression of L1 progenies by scanning through a worm culture chamber to collect data. A 10× objective was seldom utilized for the same purpose. For the observation of embryogenesis and the progression of the mitochondrial stress in the embryos, a 20× objective was found to be ideal to visualize two embryos in a single motorized stage position for time-lapse imaging (Fig. 1e).

### Fluidic experimentation protocol

**Figure 2:**
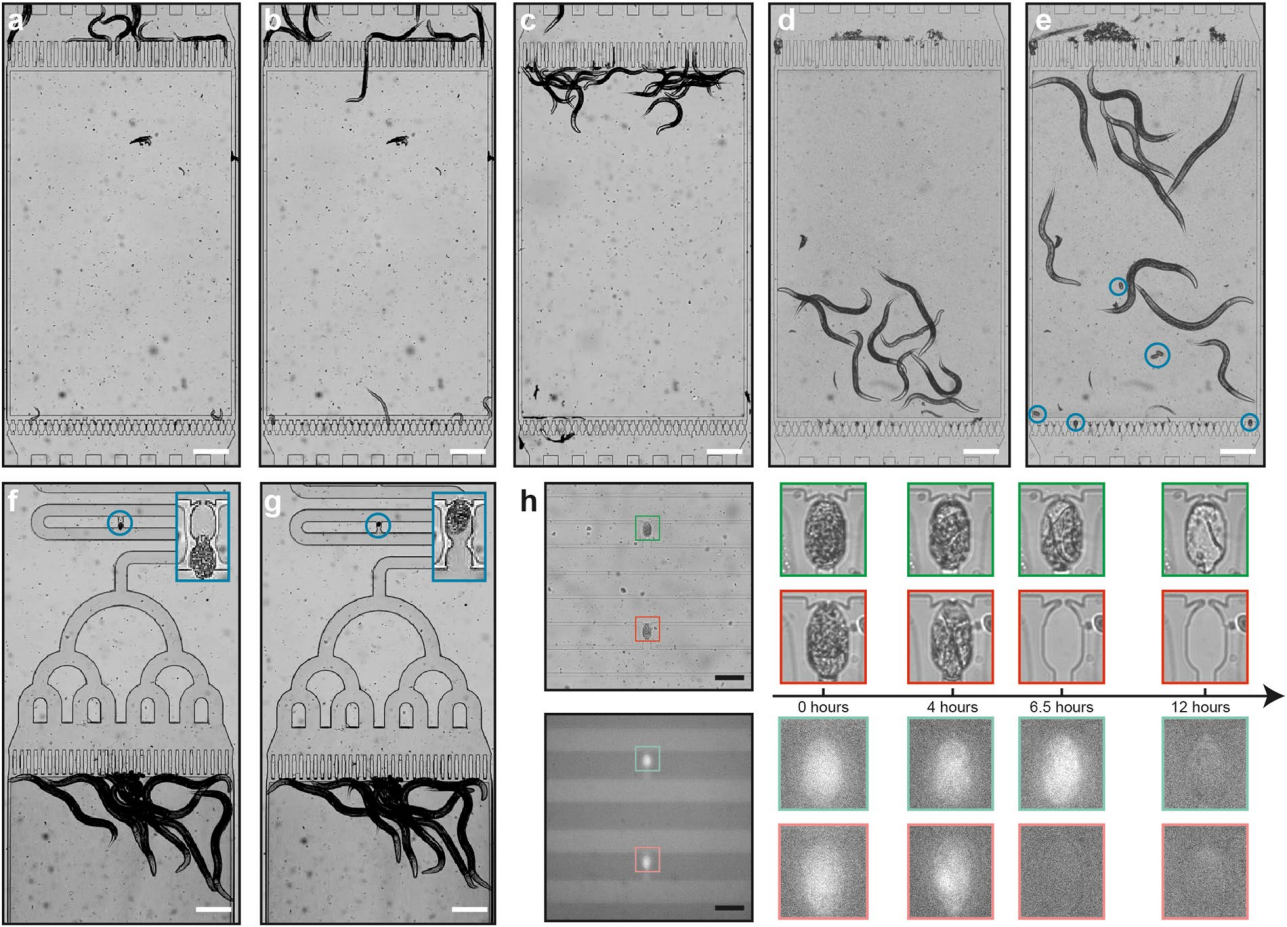
Real-time images of the experimental protocol from the beginning of an experiment until the end. (**a**) Initially, L4 larvae are collected at the entrance of the worm culture chambers. (**b**) Nematodes are pulled inside the chambers via media aspiration by a pulsed media flow, whereby they pass tightly through the constriction filters. (**c**) Any young larvae located inside the chambers are pushed towards the media outlet. (**d**) The L4 larvae suspension reservoir is replaced by an *E. coli* solution reservoir and the bacterial feeding is initiated. An image after 13 hours of the experimental onset is shown. (**e**) The feeding is stopped when all culture chambers contain first embryo progenies (displayed in blue circles). A real-time image taken after 23 hours of the experimental onset is displayed. Hereafter, embryos are (**f**) first confined in an embryo incubator and right after, (**g**) they are placed inside an embryo trap where they will be analyzed. High-resolution images of embryo incubation and trapping sites are illustrated in the blue rectangles. (**h**) Time-lapse imaging starts during 12 hours of embryo development with 10-minute intervals (both brightfield and fluorescence imaging); the sample pictures represent two embryo traps in a single motorized stage position. Scale bars (**a**–**g**): 250 µm, scale bar (**h**): 100 µm.

We created a semi-automated fluidic pipeline to reduce operator-based errors to a minimum level. Prior to the experiment initialization, all the tubing and the syringe pump were sterilized by injecting 70% ethanol solution into the whole fluidic system, after which the ethanol solution was flushed out using S-medium solution. An L4 suspension reservoir was plugged to a media outlet and the nematodes were loaded in the chip by a media injection from the media outlet towards the media inlet, collecting nematodes at the entrance filters of each worm culture chamber (Fig. 2a). A media amount of 3 µL were injected at a flow rate of 625 nL/sec with 2 second breaks from the media outlet to allow L4 larvae to pass through the filters and accommodate inside the chambers (Fig. 2b). An L4 population of 5–30 worms could be cultured thanks to the optimized fluidic protocol and the well-tuned feature size of the worm culture chambers. Depending on the goal of the user, single worms can also be distributed and cultured inside the chambers (Movie S1). Any possible early stage nematodes in a worm culture chamber and the excessive worms at the entrance of a culture chamber were washed off towards the media outlet by injecting S-medium from the media inlet at a flow rate of 104 nL/sec (Fig. 2c). The L4 suspension reservoir was then replaced by an *E. coli* solution reservoir and the bacterial solution was loaded from the media outlet at a flow rate of 156 nL/sec. At the same time, a brightfield time-lapse imaging sequence was initiated with 30-minute intervals on the worm culture chambers until the first egg release. Every 15 minutes, S-medium was supplied from the media inlet at a flow rate of 104 nL/sec to break any *E. coli* aggregate formation and then *E. coli* aspiration from the media outlet was resumed. The bacterial injection amount was kept 3.6 µL more than the S-medium supplied to ensure *E. coli* uniformity. The nematodes exhibited proper developmental behavior with this feeding protocol (see a worm culture image at 13 hours in Fig. 2d) without any bacterial clogging in the microfluidic device for the duration of the experiments. The feeding and the time-lapse sequence was terminated once worm culture chambers were occupied with first embryo progenies (Fig. 2e). In a microfluidic lane-alternating manner, pulsed S-medium flow was supplied from the media inlet (an injection amount of 20 µL and a flow rate of 2.08 µL/sec) and embryos in the culture chambers were first transferred to the embryo incubators (Fig. 2f) and subsequently confined in the embryo traps (Fig. 2g). This step typically took place in 30 minutes. The wing-shaped tip of the embryo traps (adapted from a prior work^37^) did not permit any embryo escape once the embryos were confined; however, the wing parts can be widened to support embryo recovery too. After the finalization of the embryo trapping, the *E. coli* solution reservoir was replaced with a S-medium solution reservoir and 25 µL of solution was aspirated. Hereafter, a time-lapse imaging sequence of 12 hours on two neighboring embryo traps with 10-minute intervals was initiated while providing a gentle flow of 104 nL/sec from the media outlet side (Fig. 2h). This sequence was employed in a dual brightfield and fluorescence imaging method to observe both embryogenesis and GFP expression of the embryos. Our experimental protocol is schematically shown in Supplementary Fig. S2.

### Automated and operator-based post-experiment image analysis

**Figure 3:**
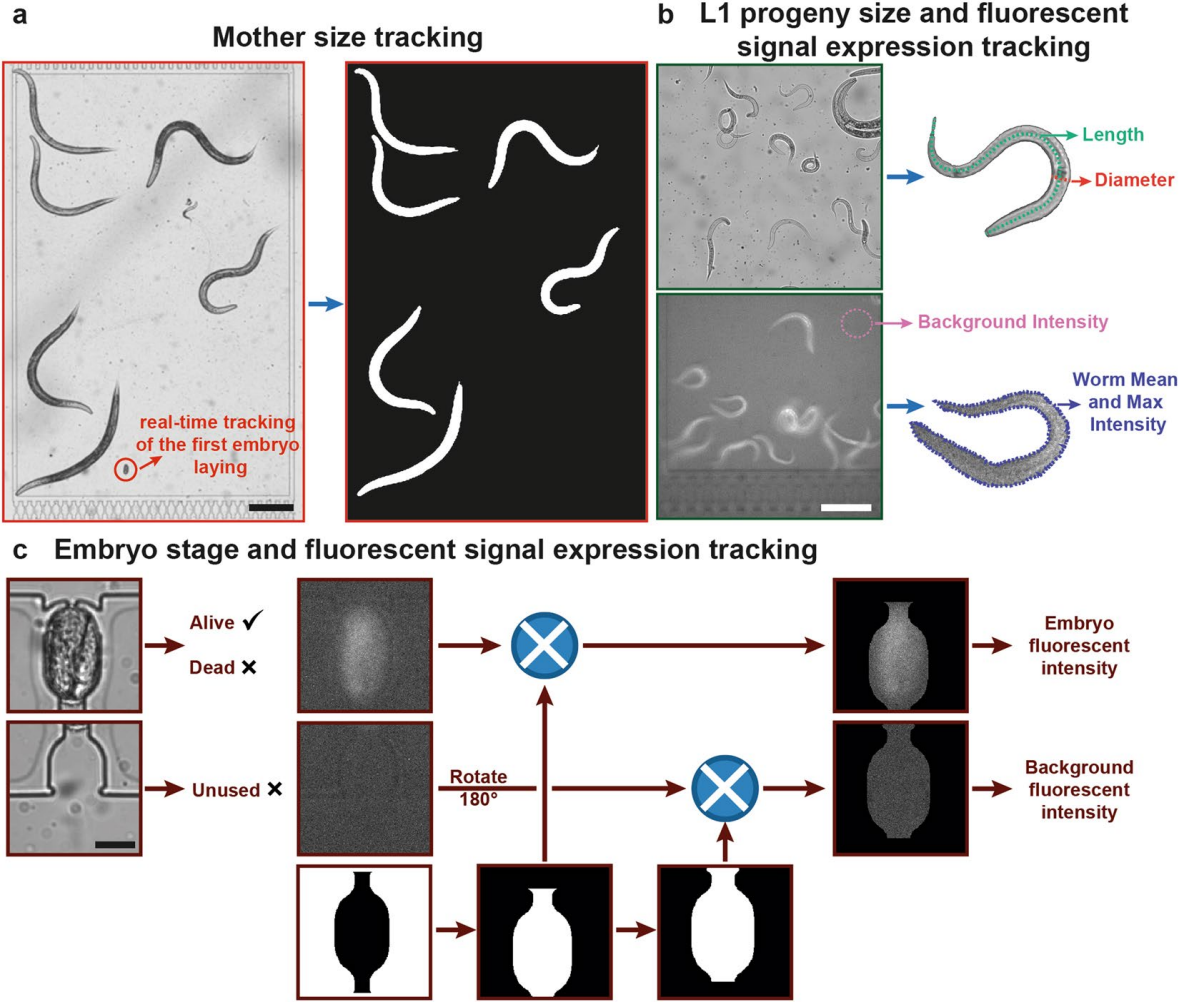
Overview of the image processing approaches employed for the phenotyping of *C. elegans*. (**a**) For the size detection of the mother worms, we capture brightfield time-lapse images with 30-minute intervals. For each captured image, the background is subtracted, standard morphological operations are applied and a binary image is created for the area detection. Operator-based observations are additionally performed to detect first embryo laying time, amount of embryos released per worm and egg release rate. Scale bar: 250 µm. (**b**) L1 progeny size and fluorescent intensity expression tracking is performed to analyze morphological properties and fluorescence intensity expression of the nematodes. Scale bar: 150 µm. (**c**) The automated embryo development algorithm is initiated to locate alive embryos. A binary mask is created and mapped on the fluorescent image patch of an alive embryo in the trap and the average fluorescent intensity expression of an embryo is extracted. A spatially shifted version of the binary mask then quantifies the background fluorescent intensity in the incubator region. The two values are divided to assess the “normalized average embryo intensity” in time. Scale bar: 20 µm.

Automated analysis can reduce the human bias and speed up the data processing time as bioanalytical studies often require large number of samples^38^. Various methods for high-throughput image acquisition and phenotyping of *C. elegans* have already been proposed^39^. In this work, we fashioned a novel approach for automated and operator-based analysis of certain phenotypes of the nematode *C. elegans*. We employed three different image analysis algorithms after the finalization of an experiment (Fig. 3). We extracted phenotypes related to the mother nematodes and L1 progenies by utilizing images acquired on the worm culture chambers and first embryo progenies by using images obtained with time-lapse imaging on the embryo traps. The total area of the mother nematodes was computed from raw brightfield images that were captured during the time-lapse imaging (Fig. 3a). Based on our previous work^27^, we applied a series of background subtraction, standard morphological operations and image binarization to isolate the nematodes from the background (see further details in Supplementary Fig. S3). The resultant image constituted of only background-isolated nematodes with binarized pixel-intensity values, over which the total sum of non-zero pixels reported the total area of all the nematodes. The normalized area data was obtained by dividing the total area by the number of worms accommodated in the associated worm culture chamber. In parallel, operator-based observations were performed to extract embryo-related phenotypes, such as the first egg laying time, released embryo amount per worm and normalized egg release rate. Another phenotype that could be obtained from the observation of the worm culture chambers was related to the L1 progenies (Fig. 3b). Operator based measurements, utilizing open-source ImageJ software, were performed by tracing a spline through L1 larvae. The measurement result of the entire spline was noted as the length of the worm, while the diameter (or the width, as the worms were assumed cylindrical) was measured as the perpendicular line to this spline fit near the worm’s vulva. Similarly, a worm was marked with a freehand line on its edges. By computing the average and the maximum pixel-intensity values within this geometry, the average (also noted as mean) and the maximum fluorescent intensity expressions, respectively, were deduced. These values were normalized by the background intensity, which was measured by computing the average pixel-intensity value on random circles drawn in the worm-free areas.

Another automation script was created to track the viability and fluorescent intensity expression of *C. elegans* embryos in the embryo traps (Fig. 3c). We first detected the location of embryo traps in an automated manner (see details in Supplementary Fig. S4a–e). After obtaining the x- and y- coordinates of the intersection between an embryo trap and an embryo incubator, we cropped 200 × 200 pixel-sized images of each part. We utilized the automation script previously created in our work^40^ to distinguish alive embryos from the dead ones in the embryo traps (by using brightfield images of embryo traps). This script, additionally, generated the twitching-to-hatching development time. Although the brightfield images of embryo incubators were unused, the fluorescent set of embryo incubator images were required to calculate an average background fluorescent intensity. We modified a binary mask from our previous work (see Supplementary Fig. S4f) and then, the downward shifted version of this mask was mapped on the fluorescent set of images on embryo traps, while the upwards shifted version was used to cover only the embryo incubators. This approach provided a faultless capture of the fluorescent intensity of embryos and the background, excluding any false intensity values resulting from the PDMS part. In the last part, the average embryo fluorescent intensity was divided by the average background fluorescent intensity to compute the “normalized average embryo intensity”.

### Parental generation development under doxycycline treatment

Mitochondria play a key role in the regulation of epigenetic mechanisms and an induction of moderate mitochondrial stress extends lifespan and promotes longevity^41–43^. Doxycycline was chosen as the mitochondrial stress inducer as it blocks mitochondrial translation, leading to an imbalance between nuclear and mitochondrial encoded proteins and inducing a mitochondrial proteotoxic stress, that subsequently will instigate the UPR^mt^ ^44^. We employed different types of doxycycline treatment for analysis of early inheritable mitochondrial stress. For some of the nematodes, we provided continuously doxycycline diluted in both *E. coli* solution and S-medium solution. This approach created a continuous doxycycline-induced stress on the mothers and embryos. We labeled this condition as Mother and Embryo Treated (MET). We also supplied doxycycline diluted in *E. coli* solution for only 15 hours for some other worms to observe if a temporary stress induction on the mother could transfer to the progeny, which was called as Only Mother Treated (OMT). For the rest, we delivered *E. coli* solution without doxycycline during the development of mother nematodes. For some of these worms, after embryo trapping, we presented doxycycline diluted in S-medium solution to detect if first embryo progenies can be stressed, while their mothers were not exposed to doxycycline. This condition was named as Only Embryo Treated (OET). Finally, we did not provide doxycycline for some worms, which was the control condition (CNT).

**Figure 4:**
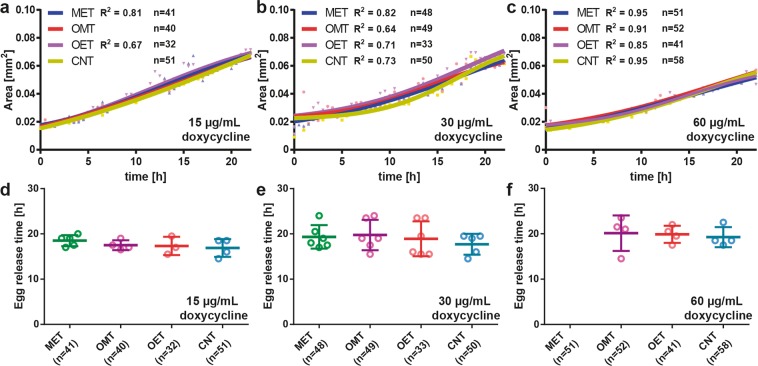
Influence of doxycycline on the development of the mother nematodes. Mothers and embryos both doxycycline-treated (MET), only mothers doxycycline-treated for 15 hours (OMT), only embryos doxycycline-treated (OET), and no doxycycline treatment (CNT) conditions are studied. (**a**–**c**) The normalized area of the mother nematodes under (**a**) 15 µg/mL, (**b**) 30 µg/mL, and (**c**) 60 µg/mL doxycycline solution. (**d**–**f**) The first egg release time of the mother nematodes under (**d**) 15 µg/mL, (**e**) 30 µg/mL and (**f**) 60 µg/mL doxycycline in *E. coli* solution. Data are expressed as mean ± SD. All measurements are based on 2 to 3 experiments for each condition. The number in “n” is the total number of worms studied for a particular condition.

First, we observed the developmental influence of doxycycline on the mother nematodes (Fig. 4). Doxycycline as an antibiotic was demonstrated to show growth-delaying effects^44^. We noticed that, during the development of mother nematodes from the L4 larval stage to the adult stage, there was no significant influence of doxycycline as a developmental lag at 15, 30 and 60 µg/mL concentrations (Fig. 4a–c). The sigmoidal fits displayed that the development trend was similar in all case studies. Thus, we concluded that doxycycline did not inhibit *E. coli* to induce caloric restriction (CR). We extracted the first egg release time – calculated after the onset of L4 larval stage – from the images of worm culture chambers. For 15 and 30 µg/mL doxycycline concentrations (Fig. 4d,e), there was a rather short delay between mother treated conditions (MET and OMT) compared to untreated conditions (OET and CNT). Surprisingly, for the MET condition at 60 µg/mL of doxycycline, the egg laying was significantly delayed (>25 hours after the onset of L4 larval stage; Fig. 4f). We waited for an additional 5 hours after the first egg release of the nematodes in the worm culture chambers of other test conditions (OMT, OET, and CNT); however, no egg laying was observed for the MET condition under 60 µg/mL doxycycline concentration within this period. In order not to miss the observation of twitching onset of OMT, OET and CNT first embryo progenies and to have all conditions within the same experimental window, we proceeded to embryogenesis observation without MET embryos. Prior research on mitochondria targeting drugs showed that at certain drug concentrations, fertility of the nematodes is strongly altered^45^. At 60 µg/mL doxycycline concentration, we observed this egg-laying alteration. As additional phenotypic parameters, we calculated the total number of embryos in the image of the time-lapse sequence that captured the first egg-laying event, and in the image of the time-lapse sequence that was captured one hour after the first egg-laying event normalized by the total number of worms accommodated (Supplementary Fig. S5a–f). We also obtained the normalized egg release rate by taking the time-derivative of the total number of embryos laid, averaged over the first two hours after the first egg-laying event (Supplementary Fig. S5g–i). We did not notice a noticeable trend for these parameters.

### Doxycycline influence on the embryogenesis of first embryo progenies

**Figure 5:**
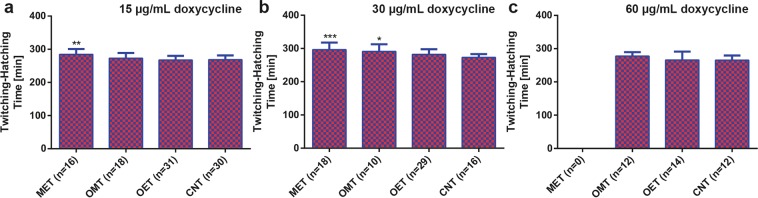
The development time of first embryo progenies released from the mother nematodes under MET, OMT, OET and CNT conditions. Twitching-to-hatching development time of the first embryo progenies under (**a**) 15 µg/mL, (**b**) 30 µg/mL and (**c**) 60 µg/mL doxycycline solution. Data are expressed as mean ± SD, *p ≤ 0.05, **p ≤ 0.01, ***p ≤ 0.001. All measurements are based on 2 to 3 experiments for each condition. The number in “n” is the total number of embryos studied for a particular condition.

After the successive trapping of *C. elegans* embryos inside the embryo traps, we could investigate the development of embryos using brightfield time-lapse imaging and automatically compute the development duration. The twitching and hatching development phases of embryos are some of the distinctive transition stages of embryogenesis^31^ and tracking the time interval between these two stages would demonstrate whether the development duration of embryos changed under doxycycline influence. We calculated the twitching-to-hatching phase duration of embryos treated with 15, 30 and 60 µg/mL doxycycline concentrations in MET, OMT, OET and CNT conditions (Fig. 5). Under 15 µg/mL doxycycline concentration, the embryos laid from the treated mothers – MET and OMT conditions – displayed rather extended development behavior (Fig. 5a). More specifically, a mother nematode treatment of 15 hours increased the twitching-to-hatching time by 4% compared to control, while continuous treatment showed that this duration increased by 6%. For treatments at 30 µg/mL doxycycline concentration, this lagging effect became more dominant, increasing the development time for MET and OMT embryos by 9 and 7%, respectively (Fig. 5b). Only 5% increase was noted for OMT embryos at 60 µg/mL concentration, while, due to the significant delay in egg laying of MET mothers, there were no embryos to calculate the development time (Fig. 5c). Additionally, for all the concentrations, there was no distinguishing developmental lag between OET and CNT embryos. It was previously reported that the inhibition of post-translational stabilization (by prohibitins) of mitochondrial respiratory enzymes could induce a developmental arrest during embryogenesis^46^. Here, we observed a similar behavior. Mitochondrial stressed mothers produced developmentally lagging embryos under all doxycycline concentrations. The effect became more prominent when the drug application time was continuous rather than being limited to 15 hours.

### Inheritance of mitochondrial stress as evidenced by the fluorescence expression of embryos

**Figure 6:**
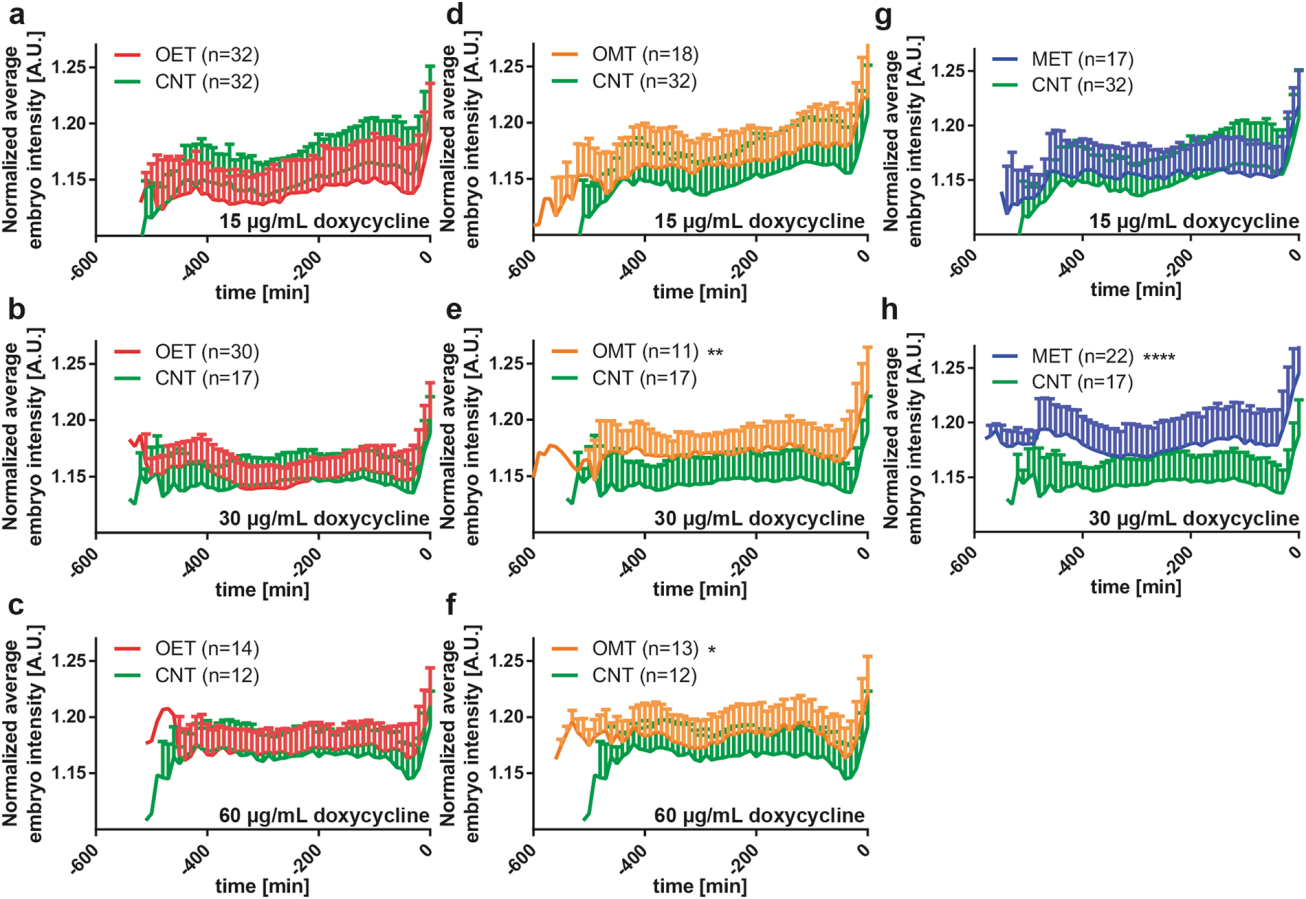
Normalized average embryo intensity profile of the first embryo progenies under MET, OMT, OET and CNT conditions and 15, 30 and 60 µg/mL doxycycline concentrations. t = 0 min is defined as the hatching event of an embryo. Normalized average embryo intensity expression of the first embryo progenies of OET and CNT conditions under (**a**) 15 µg/mL, (**b**) 30 µg/mL and (**c**) 60 µg/mL doxycycline solution. Normalized average embryo intensity expression of the first embryo progenies of OMT and CNT conditions under (**d**) 15 µg/mL, (**e**) 30 µg/mL and (**f**) 60 µg/mL doxycycline solution. Normalized average embryo intensity expression of the first embryo progenies of MET and CNT conditions under (**g**) 15 µg/mL and (**h**) 30 µg/mL doxycycline solution. Data are expressed as mean ± SD, *p ≤ 0.05, **p ≤ 0.01, ****p ≤ 0.0001. All measurements are based on 2 to 3 experiments for each condition. The number in “n” is the total number of embryos studied for a particular condition.

Certain epigenetic information is established during the sperm formation and consequently reprogrammed in *C. elegans* embryos^47^. The limit of early epigenetic transfer remains to be a motivating question however. In our case, we explored the early epigenetic inheritance of mitochondrial stress in *C. elegans*. In parallel to the brigthfield embryogenesis observations, we acquired fluorescent time-lapse images of embryos in the embryo traps. Based on our selection of *hsp-6::gfp* strain^48^, we could evaluate if the mitochondrial stress-induced mothers transferred such induction to the embryos. During our observations, we noticed that throughout the development of an embryo, the UPR^mt^ expression progressively increased over time (see a development example for a single embryo in Supplementary Fig. S6). We analyzed the normalized average fluorescent intensity expression of MET, OMT, OET and CNT first embryo progenies under 15, 30 and 60 µg/mL doxycycline concentrations (Fig. 6). All intensity data after hatching were deleted (t = 0 minutes was defined as the hatching event) and all the data were presented as the normalized average fluorescent intensity expression obtained from individual embryos. We noticed that UPR^mt^ could not be directly induced in the embryos once they were laid (OET, see Fig. 6a–c), most likely attributed to *C. elegans*’ eggshell impermeability to small molecule inhibitors^23^. However, we found a dose-dependent UPR^mt^ induction of OMT (Fig. 6d–f) and MET (Fig. 6g,h) embryos. While no UPR^mt^ induction was observed at 15 µg/mL for OMT embryos (Fig. 6d), starting from a doxycycline concentration of 30 µg/mL, the UPR^mt^ was significant (Fig. 6e,f). As expected, such a trend was also observed for MET embryos (Fig. 6g,h). Since it was already demonstrated in Fig. 6f with OMT embryos under 60 µg/mL doxycycline concentration and in Fig. 6h with MET embryos under 30 µg/mL doxycycline concentration, we would expect a significant UPR^mt^ inheritance for MET embryos under 60 µg/mL doxycycline concentration too. Previous research in this field demonstrated that information from a mother’s environment can be transferred to her offspring indeed^49^. Our results revealed that the embryos could express mitochondrial stress with the right selection of the timing and the drug concentration applied to the mothers. A doxycycline concentration of 30 µg/mL and a minimum application time of 15 hours to the mother nematodes seemed to be triggering UPR^mt^. We noticed a non-linear dose-dependent effect that was not completely unexpected. Prior research reported already a significant growth delay due to exposure of the nematodes to doxycycline at 60 µg/mL, which revealed the non-linear effect compared to lower doxycycline concentrations^36^.

### UPR^mt^ expression of L1 larvae

**Figure 7:**
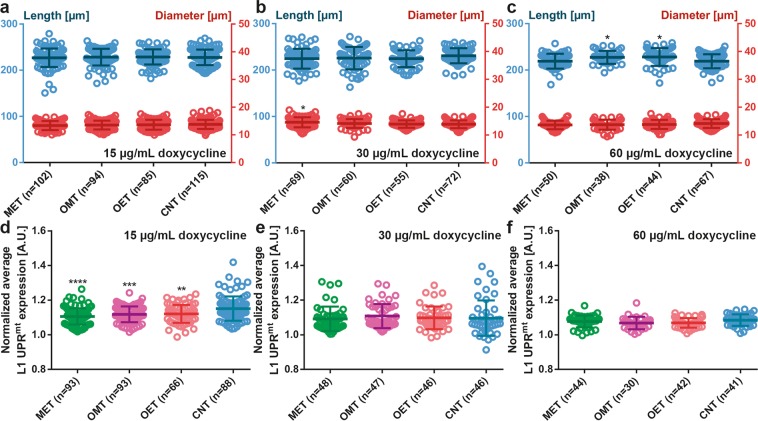
Influence of doxycycline on the size (length, diameter) and UPR^mt^ expression of L1 progenies. (**a**–**c**) Length and diameter of the L1 progenies under (**a**) 15 µg/mL, (**b**) 30 µg/mL, and (**c**) 60 µg/mL doxycycline solution. (**d**–**f**) Normalized average UPR^mt^ expression of the L1 progenies under (**d**) 15 µg/mL, (**e**) 30 µg/mL and (**f**) 60 µg/mL doxycycline solution. Data are expressed as mean ± SD, *p ≤ 0.05, **p ≤ 0.01, ***p ≤ 0.001, ****p ≤ 0.0001. All measurements are based on 2 to 3 experiments for each condition. The number in “n” is the total number of L1 progenies studied for a particular condition.

Epigenetic inheritance is frequently disappearing at the start of a new generation cycle^50^. Whether the larval stages following the embryonic stage retain this inheritance remains an interesting question. Enabled by the 10 µm wide constriction filters of the worm culture chambers, we recollected the L1 larvae after the hatching of embryos in the embryo traps, which took place typically 12 hours after the initiation of embryogenesis studies. We evaluated there if such inheritance persisted and any developmental difference was obtained under various doxycycline conditions and exposure time (Fig. 7). By monitoring the length and diameter of L1 progenies, we observed that there was no significant alteration under application of 15, 30 and 60 µg/mL doxycycline concentration (Fig. 7a–c). Of note, we noticed L1 progenies of MET condition under 60 µg/mL doxycycline concentration, indicating that eggs were laid eventually during 12 hours time-lapse imaging. At the same time, we looked at the average fluorescent intensity expression of L1 larvae (Fig. 7d–f). We noticed that for a 15 µg/mL doxycycline concentration (Fig. 7d), the application of doxycycline (MET, OMT and OET) seemed to display a slightly less UPR^mt^ expression compared to the control condition, which could however be caused by the data outliers in the control condition. This effect was absent with both 30 and 60 µg/mL doxycycline concentrations (Fig. 7e,f). In parallel, we calculated the normalized maximum UPR^mt^ expression of L1 progenies (Supplementary Fig. S7). Similar to the normalized average UPR^mt^ expression study, for all conditions, no significant trend was observed.

## Discussion

We reported here a new microfluidic platform for automated phenotyping and high-resolution studies of the nematodes and the embryos of the animal model *C. elegans*. We targeted the fabrication of a simple and easy-to-operate device. Exclusion of any on-chip active components, such as pressure valves, facilitated the fabrication protocol and solved many production-related issues. Utilizing just a single media inlet and a media outlet, our device empowers rapid loading and culture of L4 larvae, single embryo trapping, embryogenesis observation and automated phenotyping of the maternal nematodes, embryos and L1 progenies. Such a design consideration also promotes opportunities for parallel case studies, having up to 8 different conditions at the same time. Our well-tuned design features allow the culture of up to 30 nematodes in a single worm culture chamber where brightfield imaging can be performed. Typically, we culture nematodes for about 24 hours and move on to the embryo development studies. To this purpose, the fluidic serpentine contains 25 embryo traps to confine embryos accurately and enabling stable 12 hour-long embryogenesis observation using both brightfield and fluorescent imaging. Integration of three different sets of objectives in our setup permits tracking the whole dynamics of the mother and progeny development and the transition between these two states. Our microfluidic device could be further upgraded to image single cell reporters in embryos by replacing the microscope slide with a cover glass, thereby supporting the depth of field requirements of 63× oil immersion or 100× objectives.

We employed simple and repeatable fluidic commands that were deployed to a syringe pump via serial port connection. Such an approach supported repeatable experimentation and reduced the possibilities of operator-based errors by keeping the operator’s involvement during an experiment to a minimum level. An operator was involved in the experimentation only while changing the objective in use and the reservoir that was connected to a media outlet. In between experimental transitions, such as switching from the imaging of worm culture chambers to the imaging of embryo traps, a user was also required to terminate the prior stage and initiate a new one. Any possible intra-experiment variation was eliminated with this method.

We combined automated and operator-based image treatment techniques for post-experimental data analysis. We created an automated script for rapid nematode size detection. In parallel, egg-release related phenotypes, the size and the fluorescent intensity expression of L1 progenies in the worm culture chambers were obtained by operator’s observation. Utilizing an image processing script from our previous work, we could determine dead and alive embryos^40^. After locating an alive embryo, an image patch with a size of 200 × 200 pixels was cropped around the embryo traps, background-corrected and the fluorescent intensity expression of an embryo during the embryogenesis was quantified. We studied heritable transmission of mitochondrial stress by exposing *C. elegans* to doxycycline. The *hsp-6::gfp* strain reporting mitochondrial unfolded protein response was chosen as a model. We applied various doxycycline concentrations with varying application times to the mothers (OMT: 15 hours, MET: continuous) along with only doxycycline-exposed embryos (OET) and untreated (CNT) cases. Our results revealed that the doxycycline treatment from the onset of L4 larval stage did not cause any lag in the nematode growth dynamics, while causing a significant delay in the first egg laying time for MET nematodes under 60 µg/mL doxycycline condition. In addition, embryos laid by doxycycline-treated mothers showed an extended twitching-to-hatching phase duration. A doxycycline concentration equal or higher than 30 µg/mL and an application period of 15 hours resulted in stressed embryos. However, the mitochondrial stress did not persist in the L1 larval stage. We illustrated here that short-term stress inheritance in the progeny embryos could be observed in a controlled way, which possibly is an indicator of stress induction in the parental oocytes.

Our microfluidic device can be further exploited to explore additional experimental paradigms. For example, we examined already the binding of fluorescent staining agent with the eggshell under different experimental conditions using wild-type embryos (Supplementary Fig. S8). We exposed trapped embryos to well-defined concentrations and periods of a standard bleaching solution and investigated the fluorescence. Our results revealed that the intensity and the size of the fluorescent Hoechst 33342 stain aggregates inside the eggshell were significantly diminished compared to the control condition, in which embryos were not exposed to a bleach solution. Hence, we speculate that, following a bleach treatment, the eggshell eventually is thinner and less prone to molecular binding.

In summary, we showed that our new microfluidic device provided extensive automation (in terms of both fluidic handling and imaging) and enabled high-resolution and multiplexed studies, so that we could capture subtle phenotypic transitions from a mother nematode to its early progenies. We believe that many more questions in the scientific community regarding inheritance from the mother-to-progeny could be answered utilizing our platform.

## Methods

### Materials and chemicals

4-inch 550 µm thick Si wafers and AZ1512 HS photoresist were obtained from the Center of Micro- and Nanotechnology of EPFL (Lausanne, Switzerland). PDMS Sylgard 184 was acquired from Dow Corning (Wiesbaden, Germany). MicroChem SU8-3050 1 L negative photoresist was purchased from Micro Resist Technology GmbH (Berlin, Germany). Microline ethyl vinyl acetate tube with 0.51 mm inner and 1.52 mm outer diameters was obtained from Fisher Scientific (Wohlen, Switzerland). Corning microscope slides (75 mm × 38 mm) and 1*H*, 1*H*, 2*H*, 2*H*-Perfluorooctyl-trichlorosilane (FOTS) were bought from Sigma-Aldrich (Buchs, Switzerland). L-Broth bacterial culture medium was produced by adding 10 g of Bacto-tryptone, 5 g of Bacto-yeast, and 5 g of NaCl in 1 L of H_2_O. S-Basal medium was prepared by adding 5.85 g of NaCl, 1 g of K_2_HPO_4_, 6 g of KH_2_PO_4_, and 1 mL of cholesterol (5 mg/mL in ethanol) in 1 L of H_2_O. S-medium was obtained by aliquoting 0.5 mL of 1 M potassium citrate (pH 6), 0.5 mL of trace metal solution, 0.15 mL of 1 M CaCl_2_, and 0.15 mL of 1 M MgSO_4_ in 50 mL of S-Basal medium. S-Basal, L-Broth and S-medium were sterilized by autoclaving. Filtered S-medium was obtained by filtrating the S-medium solution through a syringe filter with 200 nm pore size.

### Worm and bacteria culture and embryo extraction

A single colony of *Escherichia coli* strain OP50 was used from the streak plate and injected into L-Broth medium. The injected cultures were shaken at 37 °C overnight in an incubator-shaker. Nematode growth medium (NGM) plates were then seeded with this *E. coli* OP50 food source for *C. elegans* culturing at room temperature. A similar procedure was also performed to obtain *E. coli* strain HT115 for the bacterial feeding of worms inside the microfluidic chip. The L-Broth medium of HT115 was removed after the overnight culturing by centrifuging. Freshly prepared S-medium was added and the suspension was vortexed to obtain a uniform bacterial distribution at a concentration of 4 × 10^9^ cells/mL (labeled as *E. coli* solution). A synchronized population of around 600–800 L1 worms were distributed on an NGM plate. 28 hours later, when the population reached to the L4 stage, the NGM plate was washed with S-medium and worms were suspended in an Eppendorf tube^51^. While the majority of the L4 larvae was used during the experiment, some were saved to be cultured on NGM plates for future experiments. The *hsp-6::gfp* strain was provided by the *Caenorhabditis* Genetics Center (University of Minnesota).

### Fabrication of the microfluidic chip

We employed standard soft lithography techniques for fabrication of our microfluidic chips. A 2-µm thick AZ1512 HS photoresist was patterned on a 4-inch Si wafer (corresponding to the features of the fluidic serpentine design). The wafer was etched utilizing deep reactive-ion etching (Bosch process) to obtain 40-µm deep structures. After the removal of the photoresist and surface cleaning of the wafer, a 40-µm thick SU8 layer was deposited and developed for definition of the growth chambers. The surface of the mold was functionalized with FOTS in a vacuum chamber for 12 hours to avoid sticking with the PDMS during demolding. A base-to-curing agent ratio of 10:1 PDMS mixture was dispensed on the mold and degassed for 30 minutes in a vacuum chamber, followed by a curing step at 80 °C for 2 hours. The mold was removed from the oven, the PDMS part was peeled off and 1.5 mm inlets and outlets were pierced using a biopsy punch. Both the PDMS device and a 75 mm × 38 mm glass slide were sterilized using ethanol, plasma-activated and sealed together. The bonded microfluidic chip was placed on a hotplate at 80 °C for 30 minutes to enhance the bonding, after which the chip was mounted in our experimental setup, ready to be used. A schematic illustration of the fabrication protocol can be found in Supplementary Fig. S9. We fabricated a new microfluidic chip for each experimental run.

### Availability of the design files

More information on the design files related to the microfluidic chip is available on request from the corresponding author.

### Experimental setup

A 12-port rotary valve was mounted on a Kloehn syringe pump and was connected to the microfluidic chip’s media inlets via microline ethyl vinyl acetate tubing. The microfluidic device was fixed on the motorized stage of a microscopy control system (Visitron, Puchheim, Germany). The illumination source was set at white light for brightfield microscopy and a fluorescence excitation source for fluorescence microscopy. Only brightfield imaging was utilized during the mothers’ development. Dual brightfield-fluorescent imaging was used during (i) embryogenesis observations and (ii) L1 progeny imaging. All worm culture chambers and occupied embryo traps were scanned and subsequently imaged.

### Automated image analysis

We created an automated image analysis script with Matlab (MathWorks, Natick, MA, U.S.A.) to extract *C. elegans* phenotypes throughout their development. Combined with our previously developed embryo state classification script^40^, this Matlab script served two purposes, (i) analysis of worm culture chamber brightfield images for monitoring nematode development and (ii) evaluating the fluorescent intensity expression of the alive embryos during embryogenesis.

### Statistical analysis

Data from raw images were extracted to fill an array for statistical tests using Graphpad Prism (Graphpad Software, San Diego, CA, U.S.A.). The area of the mother nematodes and the normalized average fluorescent intensity of the *hsp-6::gfp* strain were analyzed for statistical significance using Repeated Measures two-way ANOVA. The rest of the data were analyzed for statistical significance using one-way ANOVA. For the study with 15, 30 and 60 µg/mL doxycycline, we performed 2, 3 and 2 experiments for each condition, respectively. Mean values were computed to represent in graphs when measurements were repeated in multiple batches.

## Supplementary information

Supplementary Information

Movie S1

Movie S2

Movie S3

Movie S4

Movie S5

## Data Availability

The data that support the findings of this study are available on request from the corresponding author.
