# Supplementary material for: The Detection of Early Epigenetic Inheritance of Mitochondrial Stress in C. Elegans with a Microfluidic Phenotyping Platform
Source: Sci Rep. 2019 Dec 17;9:19315. doi: 10.1038/s41598-019-55979-x (PMC6917781; doi:10.1038/s41598-019-55979-x)
Supplement: Supplementary file 1 — Supplementary Information [file 41598_2019_55979_MOESM1_ESM.docx]

**Supplementary Information**

**THE DETECTION OF EARLY EPIGENETIC INHERITANCE OF MITOCHONDRIAL STRESS IN *C. ELEGANS* WITH A MICROFLUIDIC PHENOTYPING PLATFORM**

**H. B. Atakan^1^, K. S. Hof^2^, M. Cornaglia^1^, J. Auwerx^2^ and M.A.M. Gijs^1^***

^1^Laboratory of Microsystems, Ecole Polytechnique Fédérale de Lausanne, CH-1015 Lausanne, Switzerland

^2^Laboratory of Integrative Systems Physiology, Ecole Polytechnique Fédérale de Lausanne, CH-1015 Lausanne, Switzerland

*Author to whom correspondence should be addressed.

e-mail: martin.gijs@epfl.ch

**Supplementary Figures**


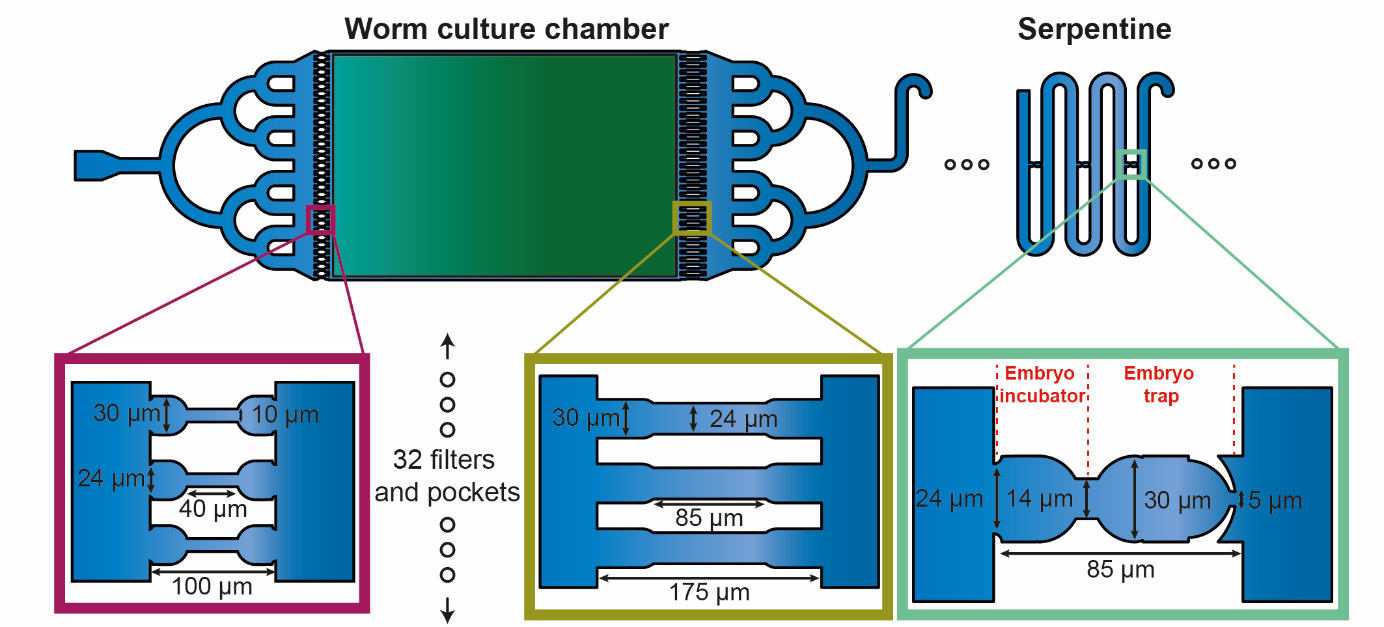


**Supplementary Figure S1. Schematic representation of a worm culture chamber (left) and a serpentine with embryo traps (right) with zooms on key features.** The worm culture chamber has a size of 1540 µm × 2385 µm and a height of 80 µm to culture up to 30 nematodes from the L4 larval stage to the adult stage. The left-hand side of the worm culture chambers consists of 32 parallel constriction filters to retain the first larval progenies after the embryo hatching (with a constriction filter width of 10 µm) in the worm culture chambers. The right-hand side of the worm culture chambers consists of 32 parallel constriction filters with a width of 24 µm in order to be tight enough to (i) selectively capture all larval stages until up to the L4 larval stage and (ii) keep embryos inside the chamber. These filters are deformable to let embryos pass only under increased flow rates (2.3 µL/sec). The serpentine is configured to act as a transfer layer for L4 larvae from the media reservoirs to the worm culture chambers and as an embryo transfer unit from the worm culture chambers to the embryo incubators. Thereafter, embryos are injected inside the embryo traps (with an entrance of 14 µm and a width of 30 µm) and studied there until hatching.


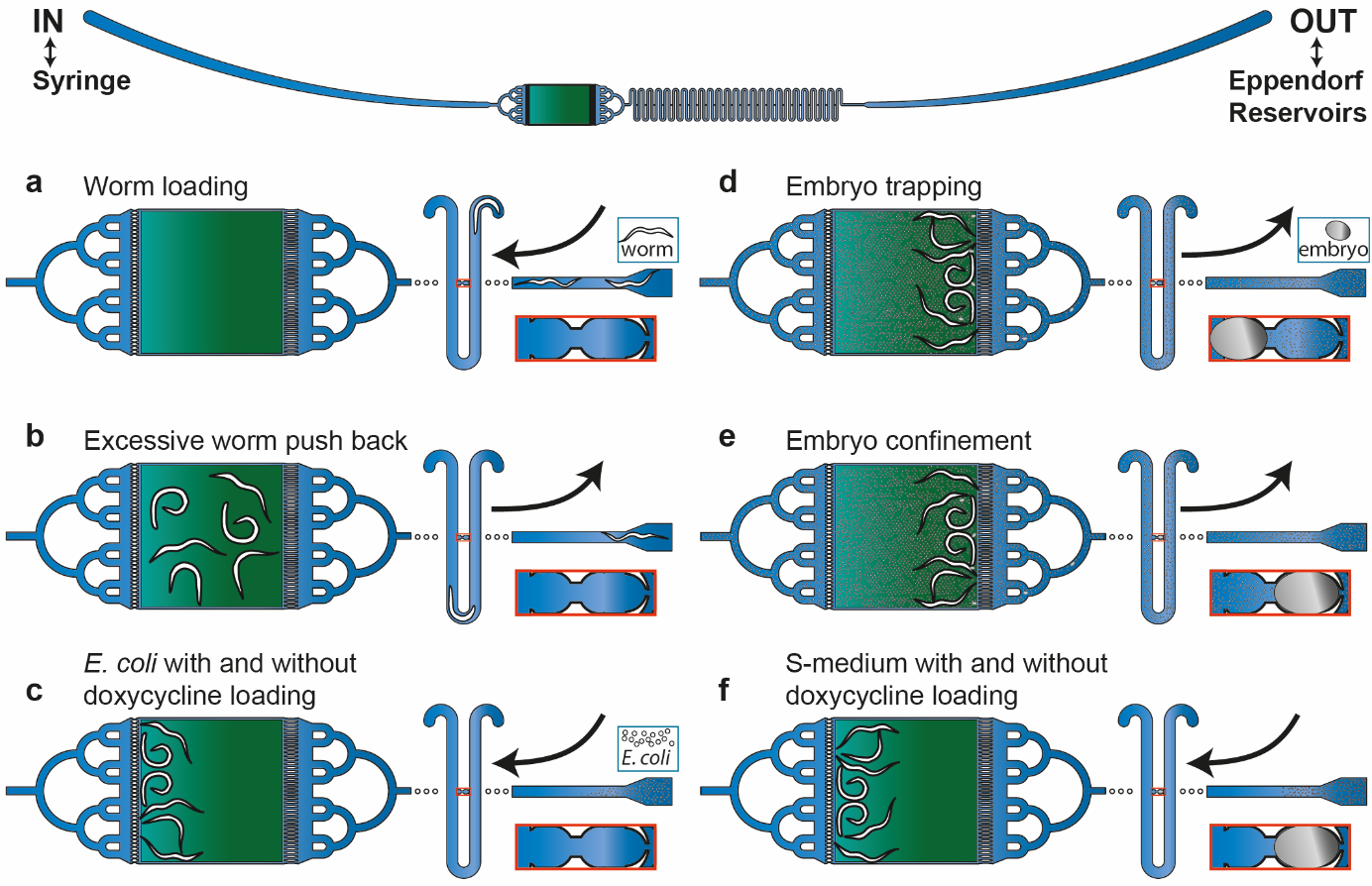


**Supplementary Figure S2. Operation principle of the microfluidic chip.** **(a)** L4 larvae are injected, through the serpentine, from the media outlet towards the media inlet. Worms are collected in front of the filter set in between the worm culture chamber and the serpentine. By applying an injection flow rate of 625 nL/sec and injection amount of 3 µL – every 2 seconds – worms are squeezed through the filters to pass inside the culture chambers. **(b)** After a worm population of 5-30 worms is located in the chamber, a flow rate of 104 nL/sec (from the media inlet towards the media outlet) is provided to retain the worms inside the worm culture chamber, while pushing back any excessive worms from the front of the filter set and from the serpentine channel. This step also removes any nematode that is younger than L4 larvae from the worm culture chambers. **(c)** The worm suspension reservoir is replaced by an *E. coli* solution reservoir and *E. coli* is aspirated from the media outlet towards the media inlet. When the first embryo laying is noticed in the worm culture chambers, the bacteria aspiration is terminated and via microfluidic injection pulses of 20 µL at 2.08 µL/sec from the media inlet towards the media outlet, embryos are **(d)** first trapped inside the embryo incubators and subsequently **(e)** enforced to a permanent more tight confinement inside the embryo traps. **(f)** After having embryo traps occupied by embryos, the *E. coli* solution reservoir is replaced by a S-medium solution reservoir. An initial media injection amount of 25 µL is used to fill the entire microfluidic lane. This is followed by the initiation of the embryo development and UPR^mt^ observation study, under application of a gentle flow of 104 nL/sec for 12 hours to preserve the solution’s concentration uniformity.


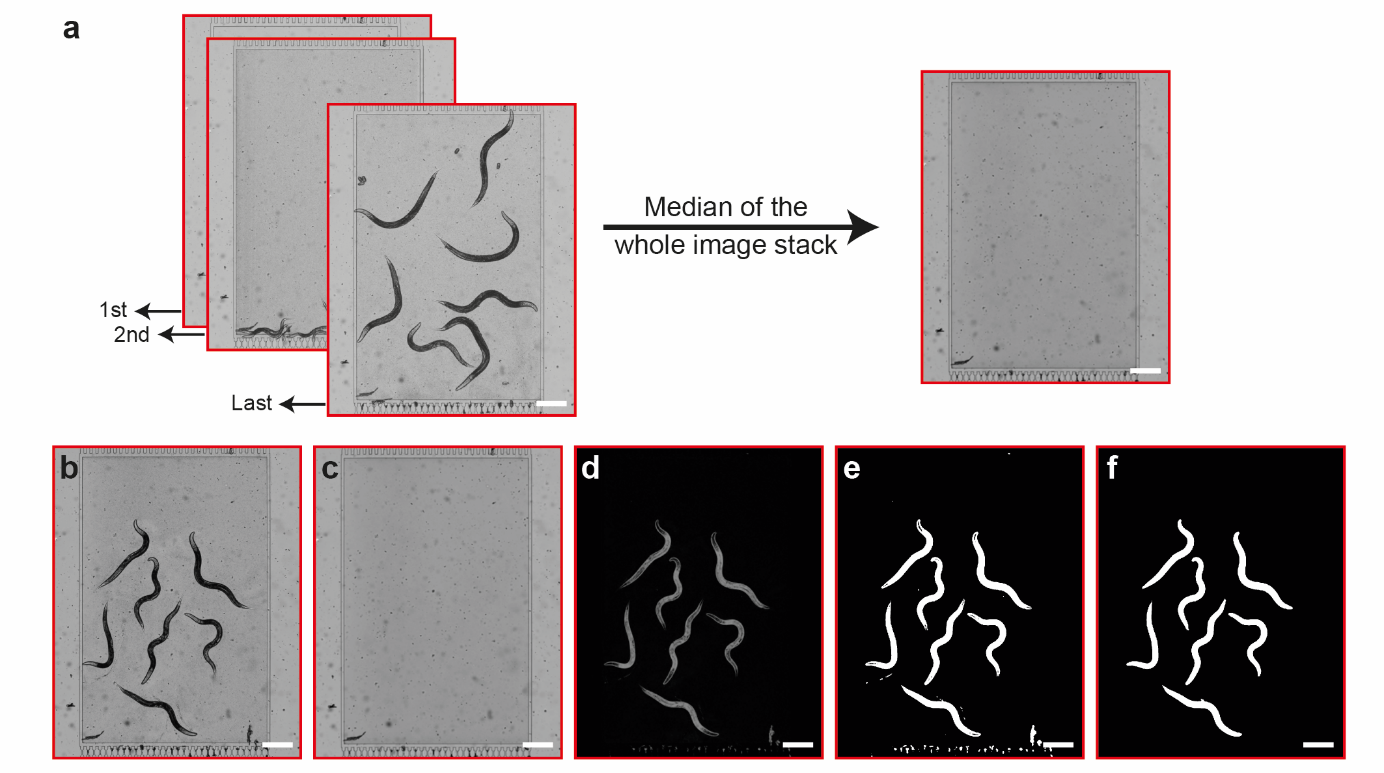


**Supplementary Figure S3. Details of the automated size detection algorithm during the worm development in the worm culture chambers.** **(a)** A time-lapse recording series with 30-minute intervals until the first egg laying of all worm culture chambers was performed. The median of the entire time-lapse series on a worm culture chamber was computed to obtain a representative background image. **(b)** Each single frame captured during the time-lapse imaging was utilized to track the worm development. **(c)** The priorly obtained background image was subtracted from each single time-lapse image **(d)**. **(e)** The subtracted image was thresholded and passed through a standard series of morphological operations. **(f)** To clean up the image, the connected components that had a size less than the 120% of the mean size of all connected components was removed and the resultant image was displayed for the normalized area assay of mother nematodes. Scale bars: 250 µm.


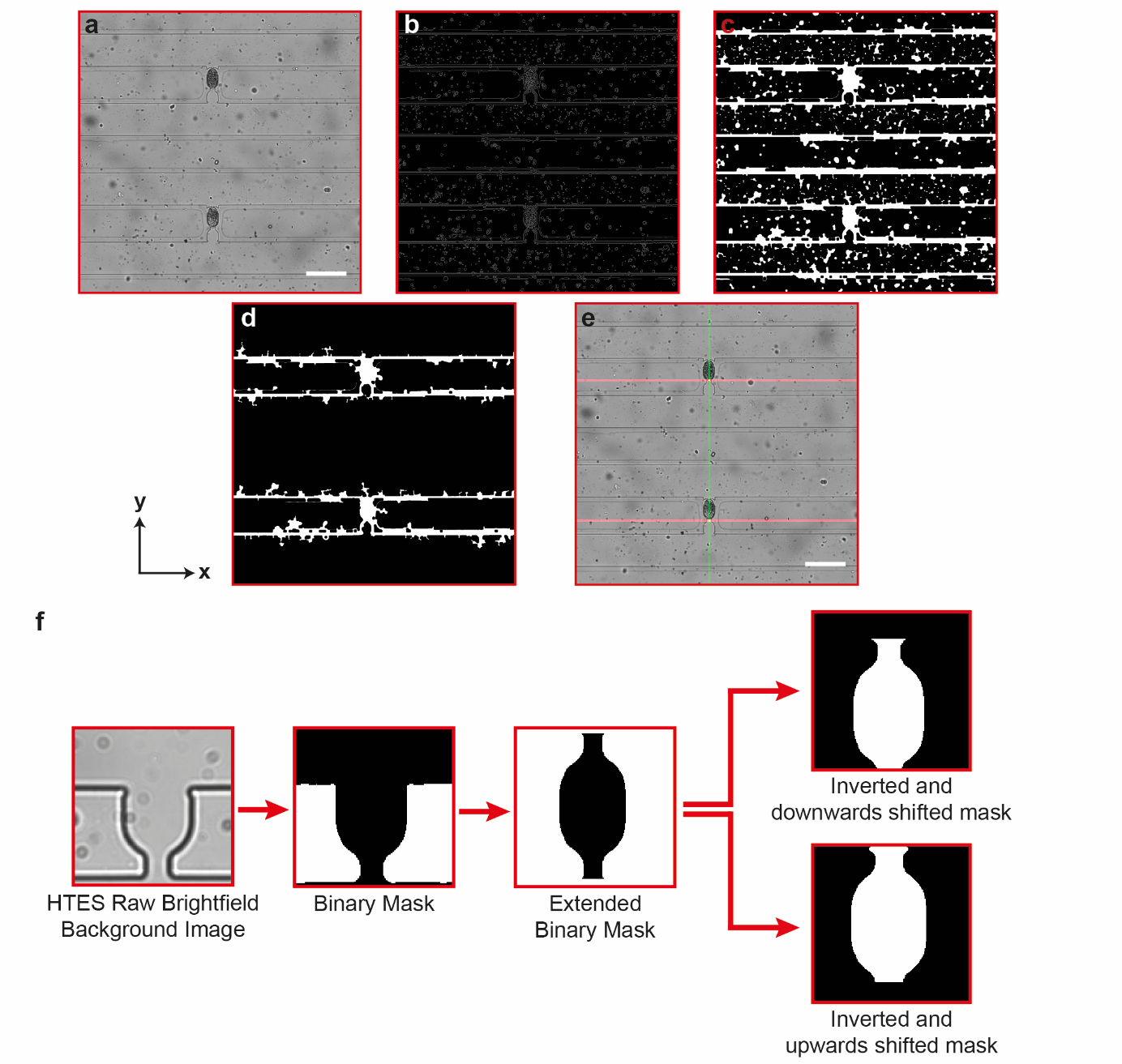


**Supplementary Figure S4. Details of the automated location detection of the embryo traps and the binary mask creation for embryos’ fluorescent intensity detection. (a)** Every 10 minutes, images of two neighboring embryo traps are captured for 12 hours with a dual brightfield-fluorescence imaging method. A sample image from the brightfield time-lapse sequence is used for the location detection of the embryo traps. **(b)** Canny edge detector is utilized to reveal the edge map information of the image of interest. **(c)** Morphological operations are applied on the resulting edge map image to reveal all embryo trap boundaries. **(d)** The largest two connected components are selected to discard the noise in the image. **(e)** In order to find the y-coordinate of the embryo traps, we sum all pixels in a row of (d), and the vicinity of the two maxima of the one-dimensional set of line-averaged pixel values allows to locate the horizontal PDMS boundaries of an embryo trap (red line). A similar approach is also performed to reveal the shared x-coordinate of the embryo traps (green line). After obtaining x- and y-coordinates for each embryo trap, 200 × 200 pixel images (corresponding to 70 × 70 µm) around each embryo trap was cropped for the subsequent embryogenesis observation and fluorescent intensity detection part of our algorithm. **(f)** An empty embryo incubator is binarized and extended by vertical duplication. During the post-processing of the embryo development analysis, this extended binary mask is inverted and shifted downwards or upwards to cover the embryo or the background regions, respectively. Scale bars: 100 µm.


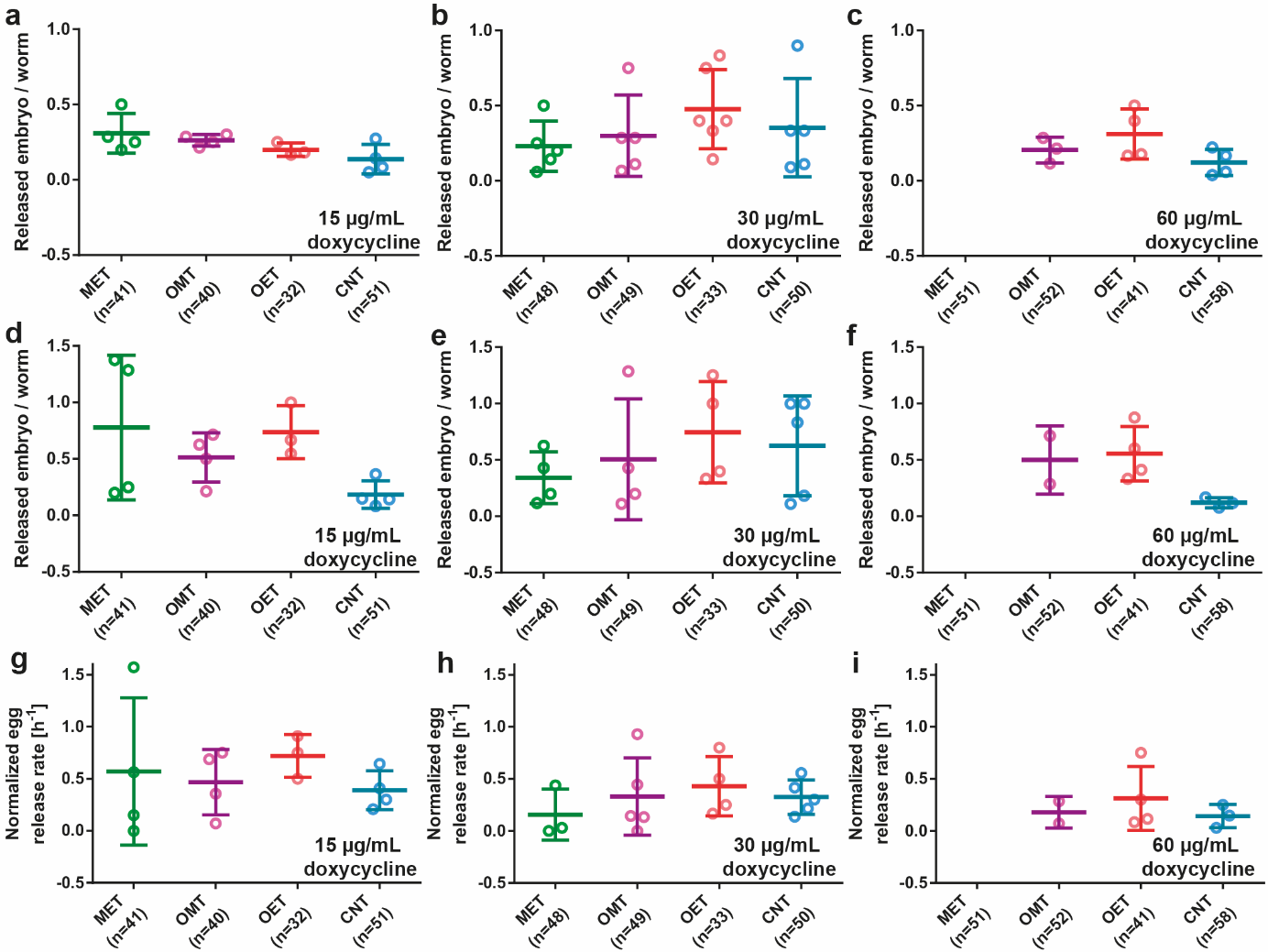


**Supplementary Figure S5. Study of the effect of doxycycline treatment on the embryo release parameters of mother nematodes. (a-c)** Total number of embryos in the image of the time-lapse sequence that captured the first egg-laying event normalized by the total number of worms accommodated in the worm culture chamber for **(a)** 15 µg/mL, **(b)** 30 µg/mL and **(c)** 60 µg/mL doxycycline solution. **(d-f)** Total number of embryos in the image of the time-lapse sequence that was captured one hour after the first egg-laying event normalized by the total number of worms accommodated in the worm culture chamber for **(d)** 15 µg/mL, **(e)** 30 µg/mL and **(f)** 60 µg/mL doxycycline solution. **(g-i)** Normalized egg release rate obtained by taking the time-derivative of the total number of embryos laid, averaged over the first two hours after the first egg-laying event for **(g)** 15 µg/mL, **(h)** 30 µg/mL and **(i)** 60 µg/mL doxycycline solution. Data are expressed as mean ± SD. All measurements are based on 2 to 3 experiments for each condition. The number in “n” is the total number of worms studied for a particular condition.


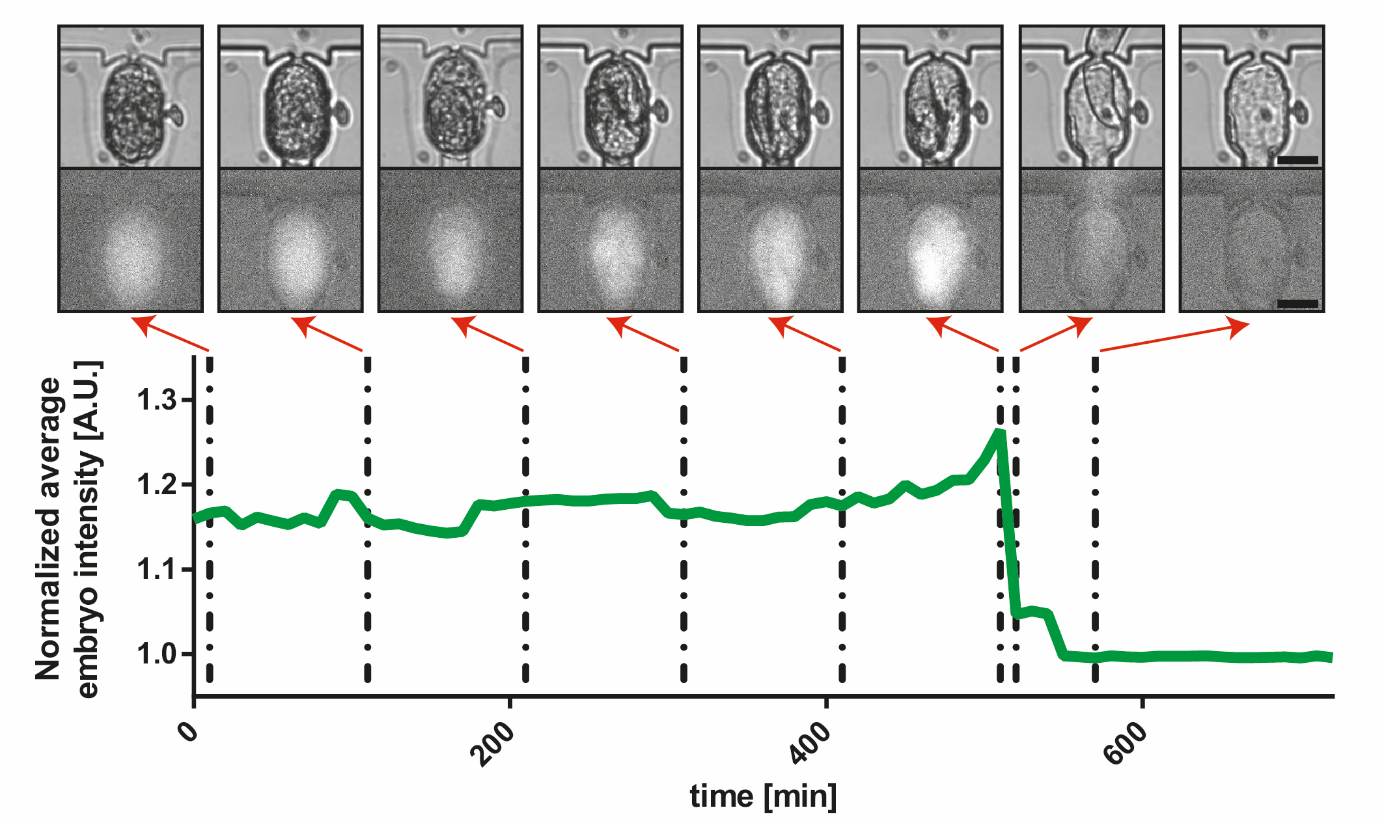


**Supplementary Figure S6. Illustrative images taken during an embryo development.** The normalized average embryo intensity during 12 hours of imaging, represented by both brightfield and fluorescent 200 × 200 pixel image patches at 10, 110, 210, 310, 410, 510, 520 and 570 minutes after the experiment initiation. Scale bars: 20 µm.


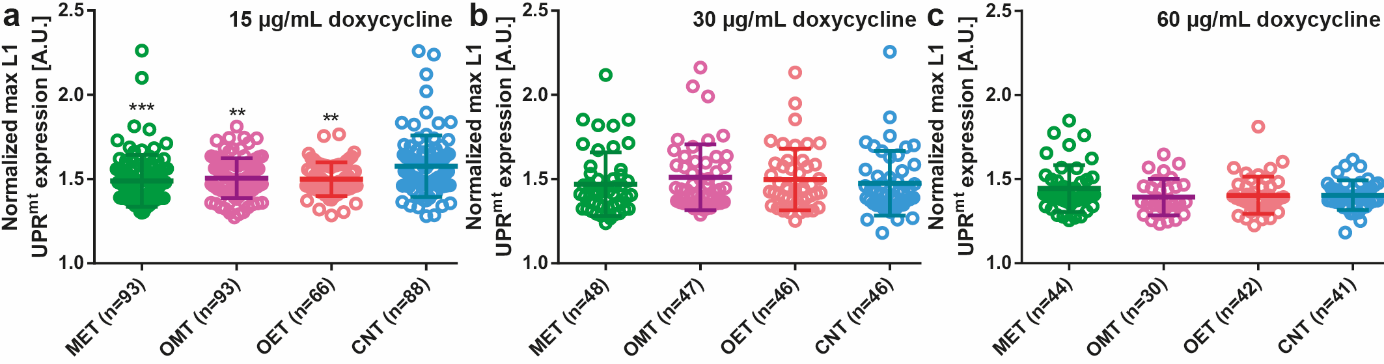


**Supplementary Figure S7. Influence of doxycycline on the maximum** **UPR^mt^ expression of L1 progenies.** Normalized maximum UPR^mt^ expression of the L1 progenies under **(a)** 15 µg/mL, **(b)** 30 µg/mL and **(c)** 60 µg/mL doxycycline solution. Data are expressed as mean ± SD, ** p ≤ 0.01, *** p ≤ 0.001. All measurements are based on 2 to 3 experiments for each condition. The number in “n” is the total number of L1 progenies studied for a particular condition.


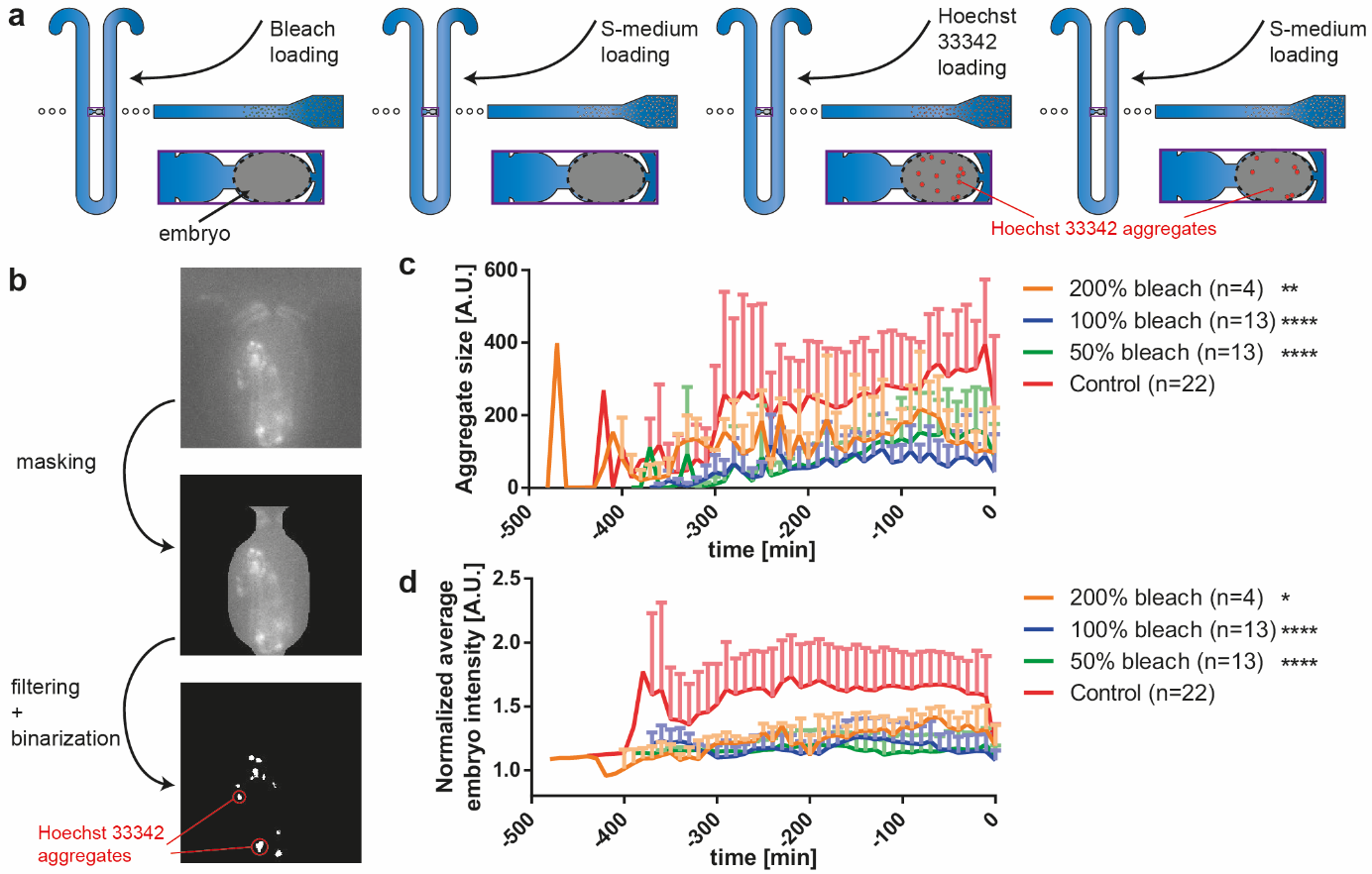


**Supplementary Figure S8.** **Influence of bleach treatment on the fluorescence of the eggshell of wild-type embryos.** After the placement of wild-type embryos in embryo traps, **(a)** bleach, S-medium, Hoechst 33342 diluted in S-medium (2 to 4 mM) and S-medium solutions were loaded each for 15 minutes, respectively. This sequence, first, compromised the outer layer of the eggshell with a bleach solution. The bleach solution was washed off with a S-medium buffer solution. This was followed by fluorescent Hoechst 33342 molecules to be loaded and observed to be bound in the eggshell. Finally, a S-medium solution was loaded to wash the serpentine and possible aggregates inside embryos. **(b)** A similar image processing approach as shown in Fig. 3 by masking the trapped embryos was utilized. The masked image was analyzed with a Laplacian of Gaussian filter (σ=10) and binarized to obtain aggregate-related phenotypes. **(c)** The aggregate size and **(d)** the normalized average embryo intensity results confirmed that the eggshell under all bleach treatment conditions were compromised enabling fluorescent material transfer. Data are expressed as mean ± SD, * p ≤ 0.05, ** p ≤ 0.01, **** p ≤ 0.0001. All measurements are based on 4 experiments for each condition. The number in “n” is the total number of embryos studied for a particular condition. 200%, 100% and 50% bleach corresponds to the combination of 0.33 mL of 4 M sodium hydroxide and 1 mL of 7-10% sodium hypochlorite solution with 1.17, 3.67 and 8.67 mL of deionized (DI) water, respectively. Control embryos were not exposed to a bleach solution.


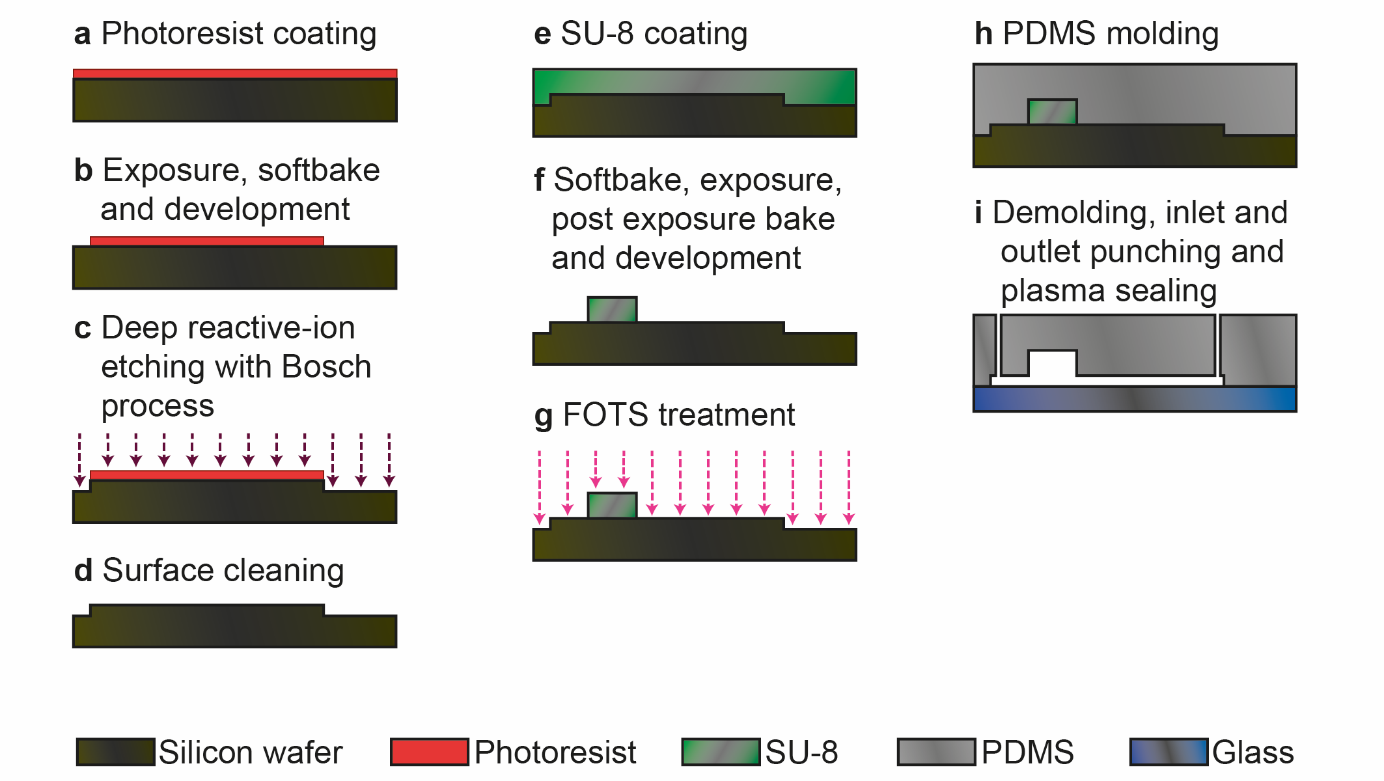


**Supplementary Figure S9.** **Details of the fabrication process of the microfluidic device.** After obtaining clean Si wafers, **(a)** a positive photoresist, AZ1512, was spin-coated till a 2 µm height. **(b)** The photoresist-coated Si wafer was exposed through a Cr mask with the design of the first layer and developed. **(c)** The wafer was etched using the deep reactive-ion etching method with the Bosch process in order to obtain an etching depth of 40 µm. **(d)** The wafer was cleaned using acetone, isopropyl alcohol and oxygen plasma treatment. **(e)** Later, SU8 (MicroChem 3050) was coated on the wafer at 80 µm height. **(f)** The wafer was soft-baked, the photoresist was exposed through a second Cr mask with the design of the second layer, and then the wafer was developed after the post exposure bake. **(g)** 1*H*,1*H*,2*H*,2*H*-Perfluorooctyl-trichlorosilane (FOTS) treatment was realized in order to facilitate PDMS demolding in the following step. **(h)** 10:1 polymer to curing agent mixture was poured on the wafer, degassed and cured for 2 hours at 80 °C. **(i)** After peeling off the PDMS mold, inlets and outlets were punched with a biopsy punch, and the PDMS chip and the glass slide were bonded by oxygen plasma treatment.

**Supplementary Movies**

**Supplementary Movie S1.** A real-time video of a L4 larva passing through the serpentine and reaching to the onset of the worm culture chamber

**Supplementary Movie S2.** A real-time video displaying the collection of L4 larvae and the selective transfer to the worm culture chamber

**Supplementary Movie S3.** A real-time video demonstrating the worm push-back and removal of young larvae

**Supplementary Movie S4.** A real-time video recorded through a 4× objective during the embryo placement and trapping

**Supplementary Movie S5.** A real-time video recorded through a 10× objective during the embryo placement and trapping
